# Comparative expression profiling in grape (*Vitis vinifera*) berries derived from frequency analysis of ESTs and MPSS signatures

**DOI:** 10.1186/1471-2229-8-53

**Published:** 2008-05-12

**Authors:** Alberto Iandolino, Kan Nobuta, Francisco Goes da Silva, Douglas R Cook, Blake C Meyers

**Affiliations:** 1 Department of Plant Pathology and College of Agricultural and Environmental Sciences Genomics Facility, University of California, One Shields Avenue, Davis, CA 95616, USA; 2 Department of Plant and Soil Sciences & Delaware Biotechnology Institute, University of Delaware, Newark, Delaware 19711, USA; 3 Monsanto, 1920 5th Street, Davis, 95616, California, USA

## Abstract

**Background:**

*Vitis vinifera *(*V. vinifera*) is the primary grape species cultivated for wine production, with an industry valued annually in the billions of dollars worldwide. In order to sustain and increase grape production, it is necessary to understand the genetic makeup of grape species. Here we performed mRNA profiling using Massively Parallel Signature Sequencing (MPSS) and combined it with available Expressed Sequence Tag (EST) data. These tag-based technologies, which do not require *a priori *knowledge of genomic sequence, are well-suited for transcriptional profiling. The sequence depth of MPSS allowed us to capture and quantify almost all the transcripts at a specific stage in the development of the grape berry.

**Results:**

The number and relative abundance of transcripts from stage II grape berries was defined using Massively Parallel Signature Sequencing (MPSS). A total of 2,635,293 17-base and 2,259,286 20-base signatures were obtained, representing at least 30,737 and 26,878 distinct sequences. The average normalized abundance per signature was ~49 TPM (Transcripts Per Million). Comparisons of the MPSS signatures with available *Vitis *species' ESTs and a unigene set demonstrated that 6,430 distinct contigs and 2,190 singletons have a perfect match to at least one MPSS signature. Among the matched sequences, ESTs were identified from tissues other than berries or from berries at different developmental stages. Additional MPSS signatures not matching to known grape ESTs can extend our knowledge of the *V. vinifera *transcriptome, particularly when these data are used to assist in annotation of whole genome sequences from *Vitis vinifera*.

**Conclusion:**

The MPSS data presented here not only achieved a higher level of saturation than previous EST based analyses, but in doing so, expand the known set of transcripts of grape berries during the unique stage in development that immediately precedes the onset of ripening. The MPSS dataset also revealed evidence of antisense expression not previously reported in grapes but comparable to that reported in other plant species. Finally, we developed a novel web-based, public resource for utilization of the grape MPSS data [1].

## Background

Grapes species (*Vitis *spp.) represent the most widely cultivated and economically important fruit crop in the world [2]. The use of grape berries includes the production of juice, fresh and dried fruit, and distilled liquor, although wine produced from cultivars of *V. vinifera *has the highest economic value of grape products. Grapevine berries are non-climacteric fruits with a characteristic double sigmoid growth curve. The initial phase of exponential berry growth (stage I) is followed by a lag phase (stage II), with growth resuming after the onset of ripening or "veraison" (stage III). Berry development is characterized by changes in numerous biological processes, including cell division and enlargement, primary and secondary metabolism, and resistance or susceptibility to abiotic or biotic stresses [3,4]. The importance of this plant species to agriculture has made the development of genomic resources a high priority. Among these resources, transcriptional profiling of important grape tissues is a practical option that may reveal transcriptional complexity and changes in this dynamic developmental system.

Massively parallel signature sequencing technology (MPSS) [5,6] is a sequence-based method for measuring gene expression. The depth of sampling provided by MPSS can identify a nearly complete inventory of transcripts in a given sample. The method is based on a unique process for parallel sequencing, which starts with the cloning of a cDNA library on 5 μm diameter microbeads; one transcript from the original RNA sample is represented on a single bead [5]. From each bead, a sequence of the 'signature' of 17 or more nucleotides is obtained by successive round of sequencing reactions [5-7]. These signatures are derived from and include the most 3' occurrence of a specific restriction enzyme site in a transcript (most often *Dpn*II, producing signatures that start with GATC) [5,6]. The output of the method is conceptually similar to a possibly more familiar method called Serial Analysis of Gene Expression (SAGE) [8]. However, the MPSS technology permits the simultaneous sequencing of millions of signatures from a given library [5]. By matching these signatures to the genome to identify specific genes, the abundance of each signature represents and measures the gene expression levels in the sample tissue. Among several published applications of this technology, we have previously conducted comprehensive transcriptional analyses of the reference plant species *Arabidopsis thaliana *and rice [7,9]. While MPSS, SAGE, and expressed sequence tags (ESTs) are all sequence-based technologies for transcriptional profiling, MPSS provides more thorough qualitative and quantitative description of gene expression due to its tremendous depth. While novel sequencing technologies, such as sequence-by-synthesis (SBS) and 454, offer deeper sequencing and longer read lengths, none have yet demonstrated consistently better results than MPSS for mRNA profiling [10].

In this report, we have measured gene expression in developing grape berries using MPSS, compared this expression profile with that provided by the current *Vitis *Unigene set [4], and we developed a novel web-based resource for utilization of the grape MPSS data. As a result of this analysis, we were able to annotate thousands of signatures matching predicted genes, quantify the expression level of these genes in the developing berries, compare the expression profiles derived from ESTs and MPSS signature frequencies, and expand the coverage of known transcripts in an important grapevine organ at a specific developmental stage. Because these data are based on sequences, they comprise a resource that will be useful for the annotation of any grape genomic sequence produced in the future.

## Results

### Analysis of the *V. vinifera *berry MPSS dataset and signature annotation

An MPSS library was constructed using RNA extracted from stage II berries (green, hard) that were sampled from field-grown *V. vinifera *cv. Cabernet Sauvignon. After cloning of the cDNA library onto beads, 17-base and 20-base signatures were generated by MPSS sequencing [5,6]. We note that these are not independent samples, in that 20-base signatures are obtained by extending previously recorded 17-base signatures by three nucleotides; due to a low failure rate at each additional base of sequencing, the raw count of sequences is lower for the 20-base data. A total of 2,635,293 17-base and 2,259,286 20-base signatures were produced that corresponded to 30,737 and 26,878 distinct sequences, respectively (Table 1A–C). This represents a discovery rate or average raw abundance value of approximately one distinctive sequence for every ~49 sequenced cDNA tags.

**Table 1:** Summary statistics of raw 17- and 20-base MPSS signatures from grape berries.

*A. Summary of sequencing results*.								
	**17-base signatures**		**20-base signatures**					
					
**Sequencing frame**	**Absolute**	**% of Total**	**Absolute**	**% of Total**				
					
2-step run	1,194,288	45.3%	1,002,346	44.4%				
4-step run	1,441,005	54.7%	1,256,940	55.6%				

*B. Single filter results*.								

	**17-base signatures**		**20-base signatures**					
					
**Reliability Filter^a^**	**Count**	**Proportion**	**Count**	**Proportion**				
					
R	17,976	58.5%	15,699	58.4%				
nR	12,761	41.5%	11,179	41.6%				
					
**Significance Filter^a^**								
					
S	16,029	52.1%	13,817	51.4%				
nS	14,708	47.9%	13,061	48.6%				
					
*C. Combined filter results*.								

	**17-base Total**		**17-base Distinct**		**20-base Total**		**20-base Distinct**	
				
**Filter categories^a^**	**Absolute**	**% of Total**	**Count**	**% of Total**	**Absolute**	**% of Total**	**Count**	**% of Total**

RS	2,550,024	96.8%	13,586	44.2%	2,187,369	96.8%	11,874	44.2%
RnS	17,862	0.7%	4,390	14.3%	14,958	0.7%	3,825	14.2%
nRS	47,346	1.7%	2443	7.9%	39,338	1.7%	1943	7.2%
nRnS	20,061	0.8%	10,318	33.6%	17,621	0.8%	9,236	34.4%

TOTAL	2,635,293	100.0%	30,737	100.0%	2,259,286	100.0%	26,878	100.0%

"Absolute" indicates the total number of successful sequencing reads, while "count" or "distinct" indicates the number of different sequences.

^a ^R = reliable, nR = non-reliable, S = significant, nS = non-significant, RS = reliable and significant, RnS = reliable and non-significant, nRS = non-reliable and significant, nRnS = non-reliable and non-significant.

Initially, to link the MPSS signatures to predicted gene annotations, all sites ("GATC") that could potentially produce an MPSS signature were identified from the available *Vitis *Unigene dataset in public databases. This comprised 14,658 contigs (1,307 from non-*vinifera Vitis *species) and 14,931 singletons (1,080 from non-*vinifera Vitis *species). All potential signatures starting with the GATC anchor sequence were extracted from both sense and antisense directions of the grape sequences. A total of 84,834 and 48,490 distinct 17-base potential signatures were identified, respectively, in contigs and singletons of this version of the *Vitis *cDNA data. When both datasets were combined, the total number of unique genomic signatures equaled 123,563. The total number of *in silico*-extracted distinct MPSS signatures is approximately six-fold lower than the 753,894 distinct "genomic" MPSS signatures reported for the completed *Arabidopsis *sequence [11], reflecting the incomplete nature of the grape EST dataset and the lack of intergenic and intron sequences.

Observed MPSS signatures were classified based on the output of "reliability" and "significance" filters [11]. The purpose of these filters is to separate high quality data, which is represented by signatures encountered above specified frequency thresholds, from background signal generated by very low abundance MPSS signatures. As with other MPSS datasets, the grape library was generated from four sequencing runs representing two sequencing frames [11]. There were two runs for each of the "two-step" and "four-step" sequencing frames. The reliability filter asks whether a signature is present in more than one sequencing run (of the four total runs); signatures observed in more than one run are considered "reliable". The significance filter identifies as "significant" only those signatures with a normalized abundance greater than three transcripts per million (TPM). The classifications of 17- and 20-base expressed signatures in terms of reliability and significance are shown in Tables 1A–C and 2; 96.8% of all MPSS signatures corresponded to the "reliable" and "significant" category, consistent with an extremely low abundance for signatures not passing the filters. This value is similar to the 97.5% reported for the *Arabidopsis *MPSS dataset [11]. Among MPSS signatures with exact sequence matches to EST contigs (Table 2A–B) and singletons (Table 2C–D), unique "reliable" and "significant" signatures represented the largest category (more than 60% of the unique signatures).

**Table 2:** Distinct MPSS signatures matching EST contigs or singletons classified based on "reliability" and "significance" filters.

*A. Filter results for 11,345 17-base signatures matching EST contigs*.		
	**Significant**	**Non-significant**
	8,012 (66.2%)	4,094 (33.8%)

**Reliable**	7,686 (63.5%)	1,734 (14.3%)
9,420 (77.8%)	6,519 contigs	1,235 contigs
**Non-reliable**	326 (2.7%)	2,360 (19.5%)
2,686 (22.2%)	632 contigs	2,276 contigs

*B. Filter results for 10,179 20-base signatures matching EST contigs*.		

	**Significant**	**Non-significant**
	7,053 (65.3%)	3,751 (34.7%)

**Reliable**	6,698 (61.1%)	1,473 (13.6%)
8,171 (75.6%)	5,830 contigs	1,490 contigs
**Non-reliable**	355 (3.3%)	2,278 (21.1%)
2,633 (24.4%)	386 contigs	2,155 contigs

*C. Filter results for 3,889 17-base signatures matching EST singletons*.		

	**Significant**	**Non-significant**
	2,700 (64.6%)	1,477 (35.4%)

**Reliable**	2,587 (61.9%)	638 (15.3%)
3,225 (77.2%)	2,423 singletons	663 singletons
**Non-reliable**	113 (2.7%)	839 (20.1%)
952 (22.8%)	127 singletons	857 singletons

*D. Filter results for 3,367 20-base signatures matching EST singletons*.		

	**Significant**	**Non-significant**
	2,318 (64.4%)	1,282 (35.6%)

**Reliable**	2,207 (61.3%)	499 (13.9%)
2,706 (75.2%)	2,096 singletons	511 singletons
**Non-reliable**	111 (3.1%)	783 (21.8%)
894 (24.8%)	128 singletons	780 singletons

Percentages refer to the total distinct signatures. Filters are described in the text, and are as defined in Meyers et al., [11]. In parts A and B, the number of EST contigs matched was out of a total of 6,430 unique contigs matched by 17-base signatures or 5,831 by 20-base signatures; signatures from different filter categories may match to the same contig (see Additional file 1A–1B and Table 3 for details). In parts C and D, the number of EST singletons matched was out of a total of 2,190 unique singletons matched by 17-base signatures or 2,097 by 20-base signatures; signatures from different filter categories may match to the same singleton (see Additional file 1A–1B and Table 3 for details).

Expressed signatures were mapped to grape EST contigs and singletons based on exact matches to the *in silico *extracted "potential signatures" (see above). A total of 5,794 and 5,407 contigs were matched by expressed reliable and significant 17-base and 20-base MPSS signatures, respectively (see Additional file 1A–1B). This represented, on average, more than 40% of all known *Vitis *sp. genes. On the other hand, only 14% of singletons in the *Vitis *sp. EST set were matched by MPSS signatures (Table 2C and 2D). The vast majority of the unmatched *Vitis sp *sequences had *in silico *potential signatures that were not detected in the MPSS data. It is possible that the corresponding genes were not expressed in this sample; alternatively, unmatched contig and singleton EST sequences may represent 5' reads of cDNA clones, and thus fail to represent 3' regions where the majority of MPSS signatures originate. The disproportionate representation of singleton ESTs among the unmatched set is consistent with this later interpretation, because singleton ESTs in the Vitis dataset are more often the product of 5' sequencing reactions.

**Figure 1:**
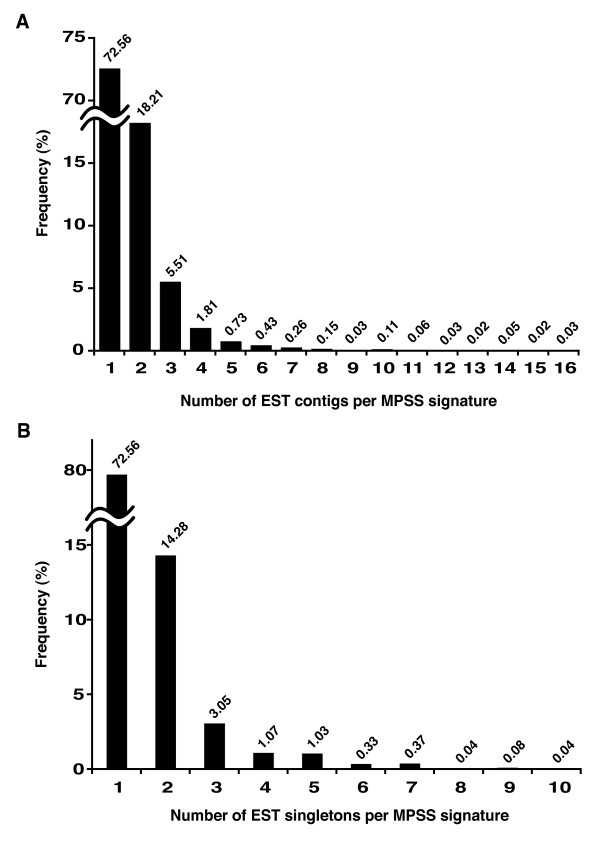
**Frequency distribution of grape ESTs matched by filtered MPSS signatures**. Reliable and significant MPSS signatures were matched to EST contigs and EST singletons. Up to 16 and 10 MPSS signatures matched to one EST contig and singleton, respectively. The proportion of the number of MPSS signatures matching to (A) EST contigs and (B) EST singletons are represented by the bar graph.

**Figure 2:**
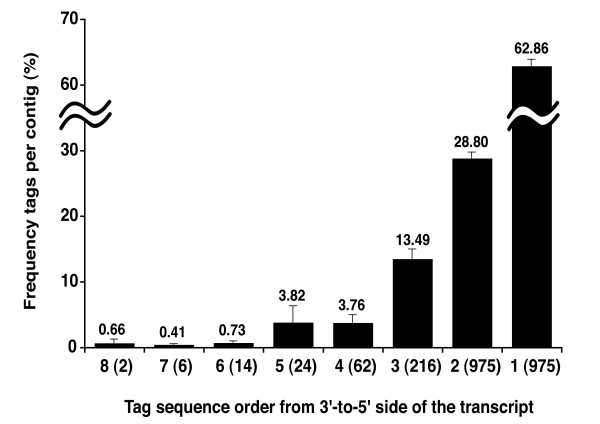
**Frequency of reliable and significant 17-mer MPSS signatures in a subset of 5'-to-3' oriented contigs**. Signatures were mapped based on their location relative to the 3' end of the EST contigs. Most signatures were found at the 3'-most *Dpn*II site, indicated as position #1 on the x-axis. However, expressed MPSS signatures were found as far 5' as eighth *Dpn*II site from the 3' end of the contig.

Most signatures matched a single contig or singleton, while ~40% matched two or more [see Additional file 1A–1B]. In excess of 70% of matched contigs and singletons showed a one-to-one assignment to a reliable and significant MPSS signature (Figure 1) [see Additional file 2]. The remaining sequences had one-to-many assignments of up to a maximum of 16 different signatures to a single contig [see Additional file 3]. Sequences of 17–20 bp are rarely duplicated by chance in unrelated genes [7] [see Additional file 4]. Instead, biological factors involving gene duplication or transcript processing may complicate the unambiguous assignment of signatures to transcripts. Thus, gene family members with high sequence similarity are likely to yield distinct transcripts containing the same signature, while the use of multiple polyadenylation sites or alternative splice site selection can yield multiple signatures from the same transcription unit. To estimate the frequency of alternative termination, a subset of 5,145 contigs was properly aligned in their 5' to 3' orientation. From this subset, 975 contigs matched by at least two MPSS signatures were identified. The abundance counts of 17-nucleotide significant and reliable MPSS signatures were transformed to relative frequency values and the location of each signature was plotted along the 3'-to-5' axis for each of the 975 contigs (Figure 2). The signature frequency per contig decreased exponentially from the 3'-to-5' direction. On average, ~70% of all signatures originate from the 3' most GATC site, while only ~29% and ~14% of signatures originate from the second and third 3' most positions (further 5'), respectively. Therefore, most of the transcripts matched by MPSS are the product of polyadenylation at the most distal of all recorded 3' sites. It is possible, however, that the MPSS signatures that did not match ESTs (contigs or singletons) are derived from longer 3' ends for which transcript sequence was not available.

### Analysis of sense-antisense expression

**Figure 3:**
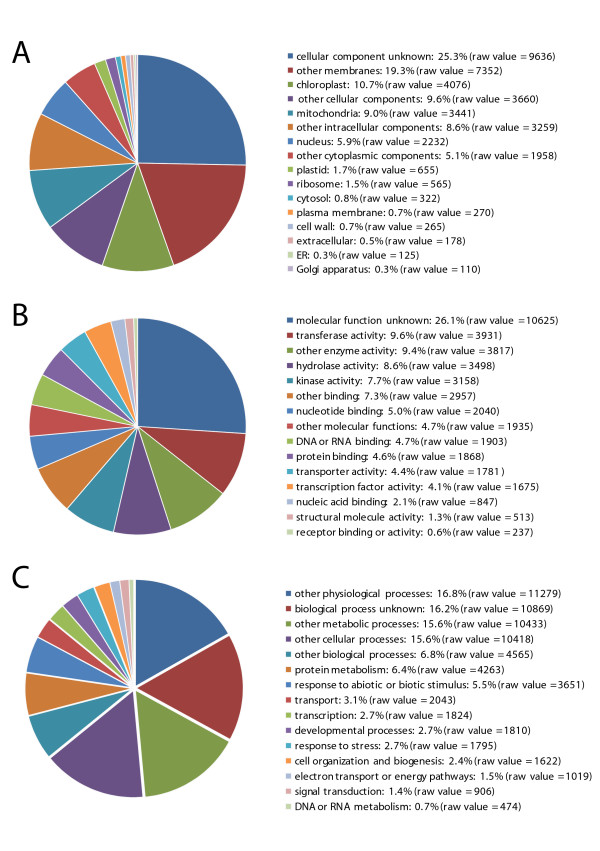
**Functional categorization of transcripts with both sense and anti-sense MPSS signatures**. EST contigs, which have both sense and anti-sense MPSS signatures, were categorized based on GO (Gene Ontology) annotation and the proportion of each category is displayed in pie-chart: (A) Cellular component, (B) Molecular function, and (C) Biological process.

Approximately 15% and 11% of the EST contigs and singletons, respectively, were matched by MPSS signatures in both sense and antisense orientations (Tables 3A–B). The MPSS signature frequencies were much higher on the sense strand for some sequences, while other sequences had higher MPSS abundances on the antisense strand [see Additional file 5]. Contigs matched in both orientations represented ~12% of the known berry transcriptome (of a total of 7,828 including contigs derived from EST sequenced and cloned from cDNA libraries other than green stage II), with the 2,891 MPSS signatures matching these contigs representing ~52% of the total MPSS abundance. It is possible that the sense-antisense transcript pairs are an important transcriptional feature which could provide a mechanism for post-transcriptional gene silencing [12] during this dynamic phase of berry development. Functional categorization of these contigs showed no particular overrepresented category (Figure 3). Moreover, none of these contigs had significant identifiable tBLASTx hits in both reading frame orientations, suggesting protein coding is a property of only one strand. It is possible that anti-sense transcripts could result from overlapping 3'UTRs of adjacent genes, or from transcription of an overlapping non-coding RNA.

**Table 3:** Matched and un-matched *Vitis *EST contig and singletons.

*A. EST contigs*.				
	**17-mer**		**20-mer**	
	
**Category^a^**	**Count**	**Freq**	**Count**	**Freq**

5' and 3'	942	6.4%	736	5.0%
5' or 3'	5,488	37.4%	5,095	34.8%

**Subtotal**	6,430	43.9%	5,831	39.8%

With tag but no match	6,875	46.9%	7,474	51.0%
Without tags	1,353	9.2%	1,353	9.2%

**Subtotal**	8,228	56.1%	8,827	60.2%

**Total**	14,658	100.0%	14,658	100.0%

*B. EST singletons*.				

	**17-mer**		**20-mer**	
	
**Category^a^**	**Count**	**Freq**	**Count**	**Freq**

5' and 3'	238	1.6%	263	1.8%
5' or 3'	1,952	13.3%	1,834	12.5%

**Subtotal**	2,190	14.9%	2,097	14.3%

With tag but no match	9,074	61.8%	9,167	62.4%
Without tags	3,667	25.0%	3,667	25.0%

**Subtotal**	12,741	86.8%	12,834	87.4%

**Total**	14,931	100.0%	14,931	100.0%

^a ^Categories are as follows: "5' and 3"' indicates contigs matched by reliable and significant (RS) tags in both sense and antisense orientation simultaneously; "5' or 3"' indicates contigs matched in their sense or antisense orientation but not both; "with tag but no match" indicates contigs with *in silico *identified GATC tag sites but without sequenced signatures (the presence of a *Dpn*II site but no matching MPSS tag, see materials and methods for details); and "without tags" indicates contigs lacking GATC sites.

### Expression profiles determined by EST and MPSS abundances

To quantify gene expression levels, we used the relative abundance of the 7,686 reliable and significant 17-base MPSS signatures from the stage II berry library. These signatures represent the most robust subset of the MPSS expression data. Although the remaining 1,734 reliable but not significant signatures were not considered in this analysis, prior analysis suggests that these signatures are likely to represent genuine transcripts expressed at very low levels [11]. The transcripts represented by these signatures may be expressed at higher levels in different specific cells or tissue layers that were not sampled.

The MPSS sequences provide an inventory of the transcript population in a given organ or tissue that can be sorted based on abundance. This data is particularly powerful when aligned with EST data from related tissues, as it allows sorting based on abundance and predicted gene function. The MPSS-matched set of 5,791 grape EST contigs are derived from a series of cDNA libraries that survey several stages of plant development, as well as responses to biotic and abiotic stress [4]. Off these, 4,753 contigs contained ESTs derived from one or more grape berry tissues, while 1,038 contigs were composed of ESTs from other grape tissues but not from berries (Table 4A). A total of 1,242 EST contigs matched by MPSS signatures were from ESTs found in only a single grape tissue; of these, 555 corresponded to berry-specific EST contigs. The remaining contigs were exclusively derived from leaves, flowers, petioles, stems, buds and even roots. The remaining 4,548 cDNA contigs and sequences were detected in two or more grape organs (Table 4A). Only three MPSS-matched EST contigs were found in all seven of the grape cDNA libraries. In a similar analysis of the EST singletons, the vast majority corresponded to transcripts previously observed exclusively in berry cDNA libraries, but only 207 were stage II berries (Table 4B). Among the contigs and singletons not previously associated with berry libraries were those derived from flower and leaf cDNA libraries. MPSS signatures provided valuable information to confirm the presence and relative transcriptional levels of transcripts. Many of these transcripts may have been previously mistakenly identified as tissue-specific based on EST data only because EST sequencing was not deep enough to detect these low abundance transcripts in different tissues. The MPSS data demonstrate that the inventory of genes in a given tissue is complex and there may be substantially more overlap in diverse tissues than previously characterized, and this can be identified only by sequencing ESTs at a very deep level.

**Table 4:** Grape ESTs derived from distinct tissue types matched by MPSS signatures (only from *Vitis vinifera*).

*A. EST contigs*.	
**Categories**	**Contigs**

Berry pre-veraison	79
Berry veraison	117
Berry post-veraison	359
**Subtotal berry**	**555**
Compound bud	87
Flower	132
Seed	1
Petiole	63
Stem	67
Leaf	332
Roots	5
**Subtotal from single organ library (excluding berry libraries)**	**687**
**Subtotal from single organ library^a^**	**1,242**

2-Organs	1,520
3-Organs	1,084
4-Organs	763
5-Organs	491
6-Organs	312
7-Organs	181
8-Organs	135
9-Organs	46
All 10 sampled organs	10
**Subtotal from multiple organ libraries^b^**	**4,542**

**Subtotal non-ESTs**	**6**

**Total matched contigs and non-EST sequences**	**5,791**

Total matched contigs not previously associated with any berry library^c^	1,038
Total matched contigs previously associated with berry libraries^f^	4,753
A. Total matched contigs from other grape berry libraries^d^	3,439
B. Total matched contigs with ≥1 EST derived from stage II berry libraries^e^	1,314

**Total unmatched contigs and non-EST sequences**	**7,567**

C. Total unmatched contigs from all libraries except (D)	6,990
D. Total unmatched contigs corresponding to UCD stage II berry libraries^h^	577

*B. EST singletons*.	

**Categories**	**Contigs**

Berry pre-veraison	177
Berry veraison	343
Berry post-veraison	389
**Subtotal berry**	**909**
Compound bud	45
Flower	231
Seed	16
Petiole	34
Stem	89
Leaf	414
Roots	49
**Subtotal from other libraries (excluding berry libraries)**	**878**

**Total matched singletons**	**1,787**

A. Total matched singletons with ≥ 1 EST derived from stage II berry libraries	207

**Total unmatched singletons**	**9,754**

B. Total unmatched singletons from all libraries except (D)	8,947
C. Total unmatched singletons corresponding to UCD stage II berry libraries	807

Only 7,686 distinct, reliable and significant, 17-base MPSS signatures were used in this analysis.

^a ^Matched contigs with at least one EST derived from berry libraries excluding those from UCD. Berry libraries in this category include multiple developmental stages and growing conditions [4].

^b ^Matched contigs with ESTs found in cDNA libraries originated from at least two or more different grapevine organs. It may or may not simultaneously include berry libraries.

^c^Indicates matched contigs assembled with ESTs not previously associated with berry libraries

^d^Indicates matched contigs assembled with ESTs not previously associated with the UCD flower-berry libraries. This number includes contigs with at least one EST derived from flower-berry libraries reported by other groups.

^e ^Indicates contigs containing at least one EST produced from the flower-berry UCD libraries [4].

^f^Corresponds to the sum of A and B.

^g^Total number of contigs and non-ESTs sequences [4] not matched by 17-mer reliable and significant tags. This value corresponds to the sum of C and D.

^h ^Contigs with at least one EST derived from SII berry libraries [4] but not matched by 17-mer reliable and significant tags.

One advantage of tag-based transcriptional profiling technologies such as ESTs, SAGE and MPSS is that the targets are not preselected prior to analysis. While the discovery rate of new transcripts using ESTs-based approaches is limited by the extent of sequencing effort and redundancy within a given cDNA library, unmatched or low abundance MPSS signatures could be used as primers for PCR based methods to expand the current set of known genes for *Vitis *[13]. There were 18,631 distinct 17-base MPSS signatures that did not match known grape EST sequences, of which 5,900 were both significant and reliable; these are most likely to represent novel genes not previously identified as transcribed or transcriptional variants. We tested this hypothesis by using available sequence of the grape genome, composed of 57,662 contigs containing 487,125,096 base pairs [14]. In total, 20,661 17-mer and 17,867 20-mer distinct MPSS signatures matched to genome contig sequences. Among these, there were 9,125 and 7,771 distinct 17-mer and 20-mer MPSS signatures that matched only genomic contigs and not ESTs. Taking the 17-mer signatures as the benchmark, the MPSS data reveal 44% more transcript diversity than recorded in the existing public EST resource.

*In silico *expression profiles resulting from EST (Table 5) and MPSS signature frequencies (Table 6) showed both differences and commonalities in the relative abundance of the top-ranked genes. For example, a common feature of both datasets is the relative high abundance of several chitinases, metallothionein-like and storage proteins, as well as a putative transcription factor and an elongation factor 1-α. On the other hand, two hexameric polyubiquitins and a plasma membrane aquaporin were among the top ranked genes based on MPSS signatures but not based on EST counts, and the opposite was true (present among top ESTs, not among MPSS signatures) for a non-specific lipid transfer protein A. A similar pattern emerges from the analysis of singleton ESTs that matched abundant MPSS signatures (Table 7). Among such singleton ESTs, there were transcripts related to cell wall modification (xyloglucan-specific fungal endoglucanase inhibitor protein and an extensin-like protein), abiotic/biotic stress factors (catalase and hydroperoxide oxidase), a eukaryotic translation initiation factor and several poorly annotated transcripts.

**Table 5:** Most highly expressed grape EST contigs in the grape berry stage II libraries, based on MPSS signature abundance.

**Contig ID**	**Signature abundance^a^**	**ESTs^b^**	**ESTs^c^**	**Total ESTs^d^**	**BlastX definition [species of closest EST match]**
1027142, 1026885, 1027113	108049	31	81	112	2S albumin precursor [Vitis vinifera]
1027101	67435	52	66	118	AF449424_1 11S globulin-like protein [Corylus avellana]
1027135, 1027103, 1027117	60308	17	36	53	Storage protein [Corylus avellana, Coffea arabica and Prunus dulcis]
1027226	46226	49	31	80	conglutin gamma [Lupinus albus]
1027222	18313	34	8	42	AF240006_1 7S globulin [Sesamum indicum]
1027379, 1026935	17310	7	8	15	plasma membrane aquaporins
1027444, 1027445, 1027446	13927	185	187	372	Endochitinase and class Ib chitinase [Galega orientalis]
1027477	13748	2	4	6	hexameric polyubiquitin
1027684, 1027685	10450	3	17	20	expressed protein [Arabidopsis thaliana]
1027454	6505	12	7	19	AF121261_1 elongation factor 1-alpha 1; EF-1-alpha1 [Lilium longiflorum]
1027543, 1027547, 1027548, 1027553, 1027554	5994	3	14	17	T06239 probable glutathione transferase (EC 2.5.1.18), 2,4-D inducible – soybean
1026907	5460	4		4	T10174 hypothetical protein – castor bean
1027486	5403	3	2	5	hexameric polyubiquitin
1027455	4782		1	1	AF121261_1 elongation factor 1-alpha 1; EF-1-alpha1 [Lilium longiflorum]
1027495	4688	2	4	6	ARF_ORYSA ADP-ribosylation factor
1027181	4668	2	1	3	TCTP_HEVBR Translationally controlled tumor protein homolog (TCTP)
1026972, 1027353, 1027133	4642	14	28	42	metallothionein-like protein type 2 [Persea americana]
1027119	4419	1	6	7	ATFP3 [Arabidopsis thaliana]
1027883	4390	1		1	LTCOR11 [Lavatera thuringiaca]
1026987	4208	36	22	58	AF281656_1 putative transcription factor [Vitis vinifera]

The expression level was determined based on the signature with the maximum normalized abundance.

^a ^The sum of abundance of matching 17-base MPSS signatures from the grape library.

^b ^ESTs derived from stage II green, hard berries

^c ^ESTs derived from stage II green, soft berries

^d ^Total signatures matching contigs present in the UC Davis berry stage II libraries.

**Table 6:** Most highly expressed grape EST contigs in the grape berry stage II libraries based on EST frequency.

**Contig ID**	**Signature abundance^a^**	**ESTs^b^**	**ESTs^c^**	**Total ESTs^d^**	**BlastX definition [species of closest EST match]**
1027444	13927	183	186	369	endochitinase
1027108	0	81	152	233	NLTA_RICCO NONSPECIFIC LIPID-TRANSFER PROTEIN A (NS-LTP A)
1027101	67435	52	66	118	AF449424_1 11S globulin-like protein [Corylus avellana]
1027142	108049	28	78	106	2S albumin precursor [Vitis vinifera]
1027226	46226	49	31	80	conglutin gamma [Lupinus albus]
1027166	0	30	44	74	putative metallothionein-like protein [Vitis vinifera]
1027085	0	13	48	61	putative metallothionein-like protein [Vitis vinifera]
1027135	60308	17	34	51	AF449424_1 11S globulin-like protein [Corylus avellana]
1027222	18313	34	8	42	AF240006_1 7S globulin [Sesamum indicum]
1026987	4208	23	14	37	AF281656_1 putative transcription factor [Vitis vinifera]
1026972	4642	10	13	23	metallothionein-like protein type 2 [Persea americana]
1027300	0	14	7	21	Transcript Antisense to Ribosomal RNA; Tar1p [Saccharomyces cerevisiae]
1027410	4208	13	8	21	AF281656_1 putative transcription factor [Vitis vinifera]
1027454	6505	12	7	19	AF121261_1 elongation factor 1-alpha 1; EF-1-alpha1 [Lilium longiflorum]
1027053	120	4	14	18	No hit
1027353	4642	4	14	18	No hit
1027684	10450	3	15	18	expressed protein [Arabidopsis thaliana]
1027078	0	3	14	17	THIH_RICCO Thioredoxin H-type (TRX-H)
1028129	3736	2	15	17	AF192486_1 omega-6 fatty acid desaturase [Sesamum indicum]
1027302	4075	13	3	16	No hit

^a ^The sum of abundance of matching 17-base MPSS signatures from the grape library.

^b ^ESTs derived from stage II green, hard berries.

^c ^ESTs derived from stage II green, soft berries.

^d ^Total signatures matching contigs present in the UC Davis berry stage II libraries.

**Table 7:** Top 20 grape EST singletons based on MPSS signature abundance.

**Singleton IDs**	**Signature abundance^a^**	**ESTs^b^**	**ESTs^c^**	**Total ESTs^d^**	**BlastX definition [species of closest EST match]**
CB346285, CB348203, CB349257, CB349340, CB350305, CB979523	108049	1	5	6	Albumin seed storage protein precursor [Juglans regia]
CB346171, CB347912, CB349205, CB349357	67435		4	4	11S globulin
CB347682	46226		1	1	Xyloglucan-specific fungal endoglucanase inhibitor protein precursor [Lycopersicon esculentum]
CB346916, CB347117, CB347160, CB347210, CB347916, CB348271, CB348487, CB348646, CB349834, CB976447	33100	1	9	10	NLTA_RICCO Nonspecific lipid-transfer protein A (NS-LTP A) (phospholipid transfer protein) (PLTP)
CB346008, CB348119, CB348425, CB348509, CB348553, CB349918	13927		6	6	Chitinases [Arabis fecunda, Glycine max, Vitis vinifera, Fragaria × ananassa, chic pea]
CB346025, CB976380	5234	1	1	2	Chitinase [Oryza sativa (indica cultivar-group)]
CB347884	4642		1	1	A34131 metallothionein I homolog – spotted monkey flower
CB348305	4208		1	1	AF281656_1 putative transcription factor [Vitis vinifera]
CB978988	2805	1		1	AF236127_1 catalase [Vitis vinifera]
CB347891	2746		1	1	IF52_NICPL Eukaryotic translation initiation factor 5A-2 (eIF-5A) (eIF-4D)
CB347634	2724		1	1	Hydroperoxide lyase [Nicotiana attenuata]
CB348030	2219		1	1	S49422 11S globulin seed storage protein – prince&apos;s feather
CB346850, CB350175	2084		2	2	2S albumin [Vitis vinifera and Helianthus annus]
CB977561	1726	1		1	AF121261_1 elongation factor 1-alpha 1; EF-1-alpha1 [Lilium longiflorum]
CB978160	1714	1		1	expressed protein [Arabidopsis thaliana]
CB977027	1343	1		1	No Hit
CB976255	1267	1		1	Expressed protein; protein id: At3g52500.1, supported by cDNA: [Arabidopsis thaliana]
CB346104, CB347550	1049		2	2	endochitinase
CB347847	983		1	1	S54157 extensin-like protein – cowpea (fragment)
CB347925, CB349273	942		2	2	S51942 prunin 2 precursor – almond (fragment)

^a ^The sum of abundance of matching 17-base MPSS signatures (reliable and significant) from the grape library.

^b ^ESTs derived from stage II green, hard berries

^c ^ESTs derived from stage II green, soft berries

^d ^Total signatures matching contigs present in the UC Davis berry stage II libraries.

Significant differences were observed in the relative abundance of contigs from EST or MPSS signature counts. While a total of 195 contigs accounted for approximately 50% of the ESTs sequenced from the two berry SII libraries, only 10 contigs matched an identical proportion of the filtered MPSS signatures. The top 20 contigs ranked based on MPSS frequency accounted for 410,925 (56.7% of all sequences matching to EST contigs), suggesting a steeper curve and perhaps lower level of diversity in MPSS data. In contrast, the 20 most frequent contigs based on EST counts represented only 29.4% of the total EST for these two libraries.

As might be expected, MPSS signatures sequenced from *V. vinifera *berries stage II also matched several non-*vinifera *EST singletons and contigs in the *Vitis *Unigene set. Although the transcriptome of the non-*vinifera *species has been minimally characterized, a comparison of the top-ranked transcripts based on MPSS signature frequency (Tables 8 and 9) showed remarkable similarities between the different species.

**Table 8:** Most highly expressed grape EST contigs from *non-vinifera *libraries based on MPSS signature abundance.

**Contig ID**	**Signature abundance^a^**	**Species^b^**	**BlastX definition [species of closest EST match]**
1025631, 1026062	17310	Vae, Vru × Var	AF141899_1 putative aquaporin PIP1-3 [Vitis berlandieri × Vitis rupestris]
1025594	13748	Vae	UQFS ubiquitin precursor – common sunflower (fragment)
1025587	6505	Vae	AF121261_1 elongation factor 1-alpha 1; EF-1-alpha1 [Lilium longiflorum]
1025940	5994	Vru × Var	T06239 probable glutathione transferase (EC 2.5.1.18), 2,4-D inducible – soybean
1025641	5460	Vae	T10174 hypothetical protein – castor bean
1026041	5403	Vru × Var	T5J8.21 polyubiquitin (UBQ14) identical to GI:166795 [N. sylvestris]
1026403	5403	Vru × Var	C17L7.6 T32N4.13 score = 526.5, E = 1.9e-154, N = 3
1025620	4688	Vae	ARF_ORYSA ADP-ribosylation factor
1025855	4642	Vru × Var	MT1_CASGL Metallothionein-like protein 1 (MT-1)
1025856	4642	Vru × Var	No hit
1025842, 1025843	4208	Vru × Var	AF281656_1 putative transcription factor [Vitis vinifera]
1026742	3463	Vru × Var	F12F1.24 putative aspartic proteinase similar to GB:AAC49730
1026015	2805	Vru × Var	T12J5.2 M4E13.140 catalase
1026595	2746	Vru × Var	F16A14.17 F7A19.4 initiation factor 5A-4 identical to initiation factor 5A-4 [A. thaliana]
1025571, 1025572	2435	Vae	T09838 chlorophyll a/b binding protein precursor – upland cotton chloroplast
1026113	2355	Vru × Var	T23E18.12 dehydrin, putative similar to dehydrin GI:975646 from [Arabidopsis thaliana]
1025893	2164	Vru × Var	No hit
1025946	1877	Vru × Var	dormancy-associated protein -related [Arabidopsis thaliana]
1025914	1840	Vru × Var	cyclophilin [Ricinus communis]
1026217	1726	Vru × Var	AF121261_1 elongation factor 1-alpha 1; EF-1-alpha1 [Lilium longiflorum]

^a ^The sum of abundance of matching 17-base MPSS signatures from the grape library.

^b ^Species abbreviations are as follows: Vae = *Vitis aestivalis; *Vru × Var = D8909-15 (*Vitis rupestris *'A. de Serres' × *Vitis arizonica*)

**Table 9:** Most highly expressed grape EST contigs from *non-vinifera *libraries based on MPSS signature abundance.

**Contig ID**	**Signature abundance^a^**	**Species**	**BlastX definition**
CB518189	17310	Vci × Vru	AF141899_1 putative aquaporin PIP1–3 [Vitis berlandieri × Vitis rupestris]
CF205324	13748	Vru × Var	polyubiquitin [Elaeagnus umbellata]
CB288827	10450	Vae	expressed protein [Arabidopsis thaliana]
CF203205	5994	Vru × Var	GTXA_TOBAC PROBABLE GLUTATHIONE S-TRANSFERASE PARA (AUXIN-REGULATED PROTEIN PARA) (STR246C PROTEIN)
CB518203	4688	Vci × Vru	ADP-ribosylation factor [Arabidopsis thaliana]
CB518174	4668	Vci × Vru	TCTP_ORYSA Translationally controlled tumor protein homolog (TCTP)
CB518217	4642	Vci × Vru	MT1_CASGL Metallothionein-like protein 1 (MT-1)
CF206203	4208	Vru × Var	AF281656_1 putative transcription factor [Vitis vinifera]
CF202582	3036	Vru × Var	No hit
CF568957	2435	Vsh	T09838 chlorophyll a/b binding protein precursor – upland cotton chloroplast
CF204795	2164	Vru × Var	phase-change related protein [Quercus robur]
CF568866	2055	Vsh	DNJH_CUCSA DnaJ protein homolog (DNAJ-1)
CB518164	1726	Vci × Vru	AF121261_1 elongation factor 1-alpha 1; EF-1-alpha1 [Lilium longiflorum]
CF568912, CF568996	1354	Vsh	RBS_FAGCR Ribulose bisphosphate carboxylase small chain, chloroplast precursor (RuBisCO small subunit)
CB518167	913	Vci × Vru	60S ribosomal protein L26 (RPL26A) [Arabidopsis thaliana]
CB289025	866	Vae	seed specific protein Bn15D1B [Brassica napus]
CB602249	846	Vae	No hit
CB289590	802	Vae	60S ribosomal protein L27a [Panax ginseng]
CB288422	779	Vae	60S ribosomal protein L19 (RPL19B) [Arabidopsis thaliana]
CB518201	714	Vci × Vru	ARF_ORYSA ADP-ribosylation factor

^a ^The sum of abundance of matching 17-base MPSS signatures from the grape library.

^b ^Species abbreviations are as follows: Vae = *Vitis aestivalis; *Vru × Var = D8909-15 (*Vitis rupestris *'A. de Serres' × *Vitis arizonica*), Vci × Vru = *Vitis cinerea *× *Vitis rupestris*, Vsh = *Vitis shutthelworthii*.

### A website for access to the grape MPSS data

To facilitate public access and utilization of the MPSS data, we developed a database and web-based interface [15]. The database and interface is a customized version of a previously described website [16]. Unlike the Arabidopsis or rice MPSS sites which utilize the complete genomic sequence of these species, our grape database focuses on EST contigs. This required the development of specialized tools and methods. For example, the incomplete nature of ESTs required a BLAST tool that would allow the user to identify the closest grape sequence to their gene of interest. The MPSS data can be accessed by entering the grape contig identifier or EST code, the MPSS signature sequence, the grape sequence of interest, or a list of contig identifiers. The data on transcriptional activity that this website provides may be used as the starting point for analyses of individual genes or gene families in grape.

## Discussion

We have explored expression patterns at a specific stage in grape berry development by comparing and combining two tag-based methods: ESTs and MPSS. Both approaches described similar patterns of transcripts abundances, although there were some clear differences perhaps associated with the methods themselves. In principle, due to deeper sequencing, the MPSS data should provide a more thorough and quantitative representation of the absolute transcript population in terms of representation and relative abundance than that from ESTs [7,11]. This is particularly true when the number of cDNA clones sequenced from any given library is low or for genes expressed at only low levels in the sampled tissues. For the EST frequency to represent the absolute transcript frequency, sequencing efforts must be large and sampling must be unbiased. The goal of achieving saturation for libraries constructed from a specific tissue may be overcome by combining library information available in public domain databases, if those resources are large enough. However, the different protocols used for library construction and EST sequencing, the lack of complete control of growing conditions, genotype and even standardized guidelines to describe a particular stage in development, makes it difficult to achieve unbiased sampling. On the other hand, MPSS analysis is also subject to bias. For example, some highly transcribed genes (based on EST frequency analysis) were unmatched by any MPSS signatures, possibly due to either the lack of a GATC site in the sequence or a technological artifact. The lack of suitable *Dpn*II sites in some *Arabidopsis *transcripts is one source of negative results in MPSS transcriptional profiles compared against other high-throughput technologies [17]. In addition, MPSS substantially underestimates expression for signatures either containing the recognition site for the Type IIS restriction endonuclease *Bbv*I (used in MPSS sequencing), or signatures containing certain four-nucleotide words in the sequencing frames [11]. The formerly high cost of tag-based methods limited biological replication as part of the experimental approach; such data would be highly desirable to determine the degree of biological variation and technical noise derived from these technologies [7]. This may be more achievable with the next generation of technologies as costs are reduced. The combined application of multiple approaches for transcriptional profiling is likely to provide the most robust determination of transcript levels.

In the grape MPSS dataset, when multiple signatures matched to one contig, these usually varied significantly in abundance. However, these data were consistent with the most abundant MPSS signature derived from the predominant form of the transcript among the ESTs [1]. An assessment of alternative transcript polyadenylation based on MPSS in diverse tissues and treatments could provide insight into this mechanism of gene regulation by identifying differentially terminated transcripts. The annotation and analysis of signatures matching multiple contigs is a more difficult task, but validation of these data could be performed by using microarrays with specifically designed probes to determine the relative expression of all matched genes, or by repeating the MPSS experiment using a different "anchoring enzyme" such as *Nla*III (CATG) instead of *Dpn*II (GATC).

The occurrence of genome-wide duplications may drive genome diversification and speciation in the plant kingdom [18]. Gene- and organ-specific silencing and unequal expression levels have been reported in upland cotton for homeologous genes resulting from whole genome polyploidization [19-21] and a similar phenomenon may be the cause of yellow-seeded commercial soybean cultivars [22]. The extent to which duplication-associated changes in gene expression may be playing a role in grapevine phenotypes is largely unknown. Due to the ancestral polyploid nature of the grape genome [23-25], duplication events leading to interactions or silencing among homeologous genes may have occurred. Evidence of extensive antisense expression was identified by comparing the ESTs and MPSS transcriptional profiling data. Initial whole transcriptome analysis in mammalian systems indicated that up to 20% of all transcripts formed sense-antisense (S/AS) pairs [26-31]. Recent analysis derived from a large scale mouse cDNA sequencing project [32] and a high resolution transcriptional map of human chromosomes [33] revealed that S/AS pairs exists for up to 72% and 50% of all mouse and human transcripts, respectively. S/AS frequencies observed in the berry transcriptome are similar to those reported in *Arabidopsis*, where approximately 22% of all known genes have tissue specific natural antisense transcript pairs [7]. Considering the unequal contribution of different genes and regions in the genome to the formation of S/AS pairs [32], whole transcriptome analysis would certainly provide a more accurate description of the extent of the phenomena in grapes than the one determined with a limited coverage of the transcriptome in this study.

Two distinct sources of native antisense expression have been identified: *cis*- and *trans*-encoded antisense [27-29]. The former correspond to transcripts derived from the opposite strand in the same genetic locus as the sense RNA. *Cis*-encoded antisense transcripts tend to have complete overlap with the sense strand forming long perfect match RNA duplexes [28]. Approximately 50% of sense-antisense pair categories in humans fell within this category [29]. *Trans*-encoded antisense transcripts derive from alternative loci and tend to have partial overlap with the sense strand of the original locus [27,28]. The function of endogenous populations of dsRNA or small RNAs in grape remain to be elucidated with more detailed experiments, and this is best performed using short-read sequencing methods [34].

Tag-based transcriptional profiling approaches provide unique advantages for the discovery of novel expressed sequences. MPSS signatures derived from a specific stage in berry developmental revealed the existence of potentially 6,345 novel transcripts in grapes. These transcripts could be more fully identified to expand the set of known and experimentally verified *Vitis *genes either by PCR-based approaches [13], or ultimately aligning the signatures with grape genomic sequence. In the absence of full genome sequence information, PCR-based approaches may become particularly important for transcripts that are difficult to identify by means of EST-based approaches due to their low copy number or technical limitations of RNA-dependent cDNA synthesis. Whole genome sequencing of the *V. vinifera *genome, combined with data-rich tag-based (ESTs and MPSS signature frequencies) and microarray-based transcriptional data will greatly contribute to our understanding of the complex relationships between genome organization, transcriptional activity, and phenotypes. Because automated genome annotation systems are both error-prone and greatly improved with the incorporation of experimental data, the EST and MPSS data will prove invaluable in the coming years for gene discovery and the annotation of genomic sequences.

## Conclusion

We have performed a complete transcriptional analysis of *V. vinifera *berries in transition to the ripening stage using MPSS combined with EST data. Approximately 30,000 distinct signatures, each representing a distinct transcript, were identified from the MPSS data and the signatures were mapped onto EST sequences. The number of MPSS signatures matching to one EST ranged from one to 16 and suggests the existence of numerous alternative transcripts in *V. vinifera*. In addition, a large set of MPSS signatures that matched to the anti-sense orientation ESTs was identified. Although the existence of antisense transcripts has been reported in many plant species, this is the first data to suggest the existence of antisense transcripts in *V. vinifera*. In addition to the signatures with EST matches, large numbers of MPSS signatures which do not match to ESTs were identified. While a small proportion could be due to sequencing errors, we believe the majority of these were mainly due to the low depth of sequence coverage in the current EST dataset; support for this interpretation derives from the fact that the proportion of signatures matching *V. vinifera *sequences was nearly doubled by incorporation of whole genome sequence data. High capacity, short read sequencing technologies, in particular next generation gigabase methods, have potential to contribute an important element to ongoing annotation of the genome sequence of *V. vinifera*. The grape MPSS data is accessible from University of Delaware MPSS website [1] and the EST data sets are available through UCDavis College of Agricultural and Environmental Sciences Genomics Facility (CGF) website [35].

## Methods

### Plant material and sampling procedures

The cDNA used for MPSS sequencing was constructed from stage II berries (green hard) sampled from field-grown *V. vinifera *cv. Cabernet Sauvignon, clone 8 vines located in the Tyree Teaching Vineyard, UC Davis, CA. Berries were sampled from multiple clusters and from different positions in individual clusters in order to ensure a representative sample. A sub-sample of berries at this stage was used to generate a cDNA library and expressed sequence tags (ESTs), as reported previously [4]. For additional details on sample handling and storage, see Goes da Silva et al., 2005.

### MPSS data generation and analysis

All MPSS was performed essentially as described previously [5,6], with the library produced and sequenced at Illumina, Inc. (formerly Solexa, Inc.; Hayward, CA). The raw and normalized MPSS data are available at University of Delaware MPSS website [1]. We compared MPSS signatures to the *V. vinifera *ESTs available at UC Davis CGF website [35] and assigned signatures to each sequence for which a perfect match was identified. The number of matches of a signature to the EST dataset was recorded as the "hits" for each signature. We merged the sequencing runs and calculate a single normalized abundance as reported earlier [11]. Contig orientation in the 5'-to-3' direction was performed using batch BLASTX search and the analysis of subject indexes of the first EST and last EST for each contig. Data analysis was conducted in MS Excel (Microsoft, Seattle, WA) and SAS V.8 statistical package (The SAS Institute, Cary, NC), or in a customized MySQL database [16] and figures in SigmaPlot version 8.0 (Systat Software Inc., San Jose, CA).

## Authors' contributions

AI performed research and analyzed data; KN performed computational research; FGdS analyzed data; DRC and BCM designed the experiments. All of the authors participated in the writing of the manuscript.

## Supplementary Material

Additional file 1 Filtered MPSS signatures matching to grape EST contigs. Table A: 17-mer signatures. Table B: 20-mer signatures Click here for file

Additional file 2 Number of MPSS signatures matching to contigs and singletons. All the unique signatures (both 17 and 20-mer) were categorized into the following eight categories: Reliable (R), not Reliable (nR), Significant (S), not Significant (nS), Reliable and Significant (RS), Reliable but not Significant (RnS), not Reliable but significant (nRS), and not Reliable and not Significant (nRnS). The number and the frequency of the signatures in each category were identified in both sense and antisense orientation. Panel A: 17-mer MPSS signatures matched to EST contigs. Panel B: 20-mer MPSS signatures matched to EST contigs. Panel C: 17-mer MPSS signatures matched to EST singletons. Panel D: 20-mer MPSS signatures matched to EST singletons. Click here for file

Additional file 3 Iandolino. Frequency distribution of grape ESTs matched by MPSS signatures. The tables in this file show the frequency of MPSS signatures matching to ESTs. The frequency ranges from one to 16 for EST contigs (panel A and B) and 1 to 10 for EST singletons (panel C and D). Data in each table are categorized based on the filters we used to sort MPSS signatures: RS, reliable and significant; RnS, reliable but non-significant; nRS, non-reliable but significant; nRnS, non-reliable and non-significant. Panel A: 17-mer MPSS signatures matched to EST contigs. Panel B: 20-mer MPSS signatures matched to EST contigs. Panel C: 17-mer MPSS signatures matched to EST singletons. Panel D: 20-mer MPSS signatures matched to EST singletons. Click here for file

Additional file 4 Example of a grape EST contigs matched by multiple MPSS signatures. One of the EST contigs with 3 MPSS signatures matches is shown. This contig (CTG1027770) has similarity to "putative transcription factor BTF3-like mRNA". Panel A shows all the MPSS signatures identified in this contig, with the abundance level and its coordinate on the contig. Panel B displays the sequence of this contig and all the sense MPSS signatures from panel A are indicated in blue. Uppercase letters indicate the predicted ORF, while lowercase letters indicate the predicted UTRs. The position of the most abundant signature (#2) is consistent with the most-3' *Dpn*II site, the position measured by MPSS. Other signatures may result from signatures resulting from other transcripts, alternative polyadenylation or incomplete digestion during the construction of the MPSS library. Click here for file

Additional file 5 Occurrence of identical MPSS signatures in related and unrelated contigs. The example of MPSS signatures with multiple hits to EST contigs is shown. In this particular example, the MPSS signature "GATCAAGACTGATGAAA" (displayed in red) was identified in three EST contigs where two of them have the same annotation and the third is different. The most closely related Arabidopsis homolog along with its BLAST expected value is list at the beginning of each coding sequence. Click here for file

Additional file 6 EST contigs with expressed sense and antisense MPSS signatures. All the EST contigs with MPSS signatures matching to both sense and antisense orientation are displayed. Each contig was BLASTed against Arabidopsis annotation version 5 (TIGR5) and the potential function of the contigs was listed under "blastdef" along with the gene ID ("ginumber") and the BLAST expected value ("evalue"). The contigs originated from two different EST corrections (Stage II berry GH and GS) that derived from various *Vitis *species. The EST ID numbers for GH and GS, as well as the species name, are listed under "Berry SII-GH", "Berry SII-GS", and "SPECIES". Click here for file
