# Supplementary material for: Comparative expression profiling in grape (Vitis vinifera) berries derived from frequency analysis of ESTs and MPSS signatures
Source: BMC Plant Biol. 2008 May 12;8:53. doi: 10.1186/1471-2229-8-53 (PMC2423195; doi:10.1186/1471-2229-8-53)
Supplement: Additional file 1 — Filtered MPSS signatures matching to grape EST contigs. Table A: 17-mer signatures. Table B: 20-mer signatures [file 1471-2229-8-53-S1.doc]

**Additional File 1A**

**Table 1. Filtered MPSS signatures matching to grape EST contigs.**

A. 17-base signatures

| **Signatures** | **All signatures** | | **RS** | | **nRnS** | | **RnS** | | **nRS** | |
| --- | --- | --- | --- | --- | --- | --- | --- | --- | --- | --- |
|  | **Contigs** | **%** | **Contigs** | **%** | **Contigs** | **%** | **Contigs** | **%** | **Contigs** | **%** |
| 1 | 4279 | 59.16 | 4282 | 73.90 | 1710 | 84.65 | 1379 | 87.39 | 316 | 99.06 |
| 2 | 1721 | 23.79 | 1020 | 17.60 | 247 | 12.23 | 170 | 10.77 | 3 | 0.94 |
| 3 | 630 | 8.71 | 287 | 4.95 | 48 | 2.38 | 23 | 1.46 |  |  |
| 4 | 293 | 4.05 | 97 | 1.67 | 9 | 0.45 | 4 | 0.25 |  |  |
| 5 | 133 | 1.84 | 45 | 0.78 | 6 | 0.30 | 2 | 0.13 |  |  |
| 6 | 64 | 0.88 | 23 | 0.40 |  |  |  |  |  |  |
| 7 | 39 | 0.54 | 10 | 0.17 |  |  |  |  |  |  |
| 8 | 26 | 0.36 | 9 | 0.16 |  |  |  |  |  |  |
| 9 | 6 | 0.08 | 3 | 0.05 |  |  |  |  |  |  |
| 10 | 16 | 0.22 | 7 | 0.12 |  |  |  |  |  |  |
| 11 | 6 | 0.08 | 3 | 0.05 |  |  |  |  |  |  |
| 12 | 5 | 0.07 | 2 | 0.03 |  |  |  |  |  |  |
| 13 | 5 | 0.07 | 1 | 0.02 |  |  |  |  |  |  |
| 14 | 6 | 0.08 | 3 | 0.05 |  |  |  |  |  |  |
| 15 | 2 | 0.03 | 1 | 0.02 |  |  |  |  |  |  |
| 16 | 1 | 0.01 | 1 | 0.02 |  |  |  |  |  |  |
| 18 | 1 | 0.01 |  |  |  |  |  |  |  |  |
| **Total** | 7233 | 100% | 5794 | 100% | 2020 | 100% | 1578 | 100% | 319 | 100% |

B. 20-base signatures

| **Signatures** | **All signatures** | | **RS** | | **nRnS** | | **RnS** | | **nRS** | |
| --- | --- | --- | --- | --- | --- | --- | --- | --- | --- | --- |
|  | **Contigs** | **%** | **Contigs** | **%** | **Contigs** | **%** | **Contigs** | **%** | **Contigs** | **%** |
| 1 | 4209 | 62.29 | 4078 | 75.42 | 1595 | 85.89 | 940 | 90.56 | 461 | 97.05 |
| 2 | 1534 | 22.70 | 915 | 16.92 | 214 | 11.52 | 88 | 8.48 | 13 | 2.74 |
| 3 | 539 | 7.98 | 243 | 4.49 | 35 | 1.88 | 9 | 0.87 | 1 | 0.21 |
| 4 | 249 | 3.69 | 78 | 1.44 | 10 | 0.54 | 1 | 0.10 |  |  |
| 5 | 86 | 1.27 | 35 | 0.65 | 3 | 0.16 |  |  |  |  |
| 6 | 52 | 0.77 | 21 | 0.39 |  |  |  |  |  |  |
| 7 | 33 | 0.49 | 11 | 0.20 |  |  |  |  |  |  |
| 8 | 18 | 0.27 | 7 | 0.13 |  |  |  |  |  |  |
| 9 | 7 | 0.10 | 2 | 0.04 |  |  |  |  |  |  |
| 10 | 8 | 0.12 | 7 | 0.13 |  |  |  |  |  |  |
| 11 | 9 | 0.13 | 2 | 0.04 |  |  |  |  |  |  |
| 12 | 5 | 0.07 | 2 | 0.04 |  |  |  |  |  |  |
| 13 | 2 | 0.03 | 1 | 0.02 |  |  |  |  |  |  |
| 14 | 3 | 0.04 | 3 | 0.06 |  |  |  |  |  |  |
| 15 | 2 | 0.03 | 1 | 0.02 |  |  |  |  |  |  |
| 16 | 1 | 0.01 | 1 | 0.02 |  |  |  |  |  |  |
| 18 | 1 | 0.01 |  |  |  |  |  |  |  |  |
| Total | 6757 | 100% | 5407 | 100% | 1857 | 100% | 1038 | 100% | 475 | 100% |

Data are sorted based on the number of contigs matched by each type of signature. “Signatures” refers to the number of distinct signatures that matched to a contig. RS, “reliable” and significant; RnS, “reliable” but non-significant; nRS, non-”reliable” but significant; non-”reliable” and non-significant.
