# Supplementary material for: Comparative expression profiling in grape (Vitis vinifera) berries derived from frequency analysis of ESTs and MPSS signatures
Source: BMC Plant Biol. 2008 May 12;8:53. doi: 10.1186/1471-2229-8-53 (PMC2423195; doi:10.1186/1471-2229-8-53)
Supplement: Additional file 3 — Iandolino. Frequency distribution of grape ESTs matched by MPSS signatures. The tables in this file show the frequency of MPSS signatures matching to ESTs. The frequency ranges from one to 16 for EST contigs (panel A and B) and 1 to 10 for EST singletons (panel C and D). Data in each table are categorized based on the filters we used to sort MPSS signatures: RS, reliable and significant; RnS, reliable but non-significant; nRS, non-reliable but significant; nRnS, non-reliable and non-significant. Panel A: 17-mer MPSS signatures matched to EST contigs. Panel B: 20-mer MPSS signatures matched to EST contigs. Panel C: 17-mer MPSS signatures matched to EST singletons. Panel D: 20-mer MPSS signatures matched to EST singletons. [file 1471-2229-8-53-S3.pdf]

Additional File 3: Example of a grape EST contig matched by multiple MPSS signatures.

Contig ID: CTG1027770
Annotation: putative transcription factor BTF3-like mRNA

#	Signature	Abundance	Strand	Position
1	GATCTCCTCAAACAAAG	6	+	316
2	GATCTTGTTGCTGGGCA	1367	+	480
3	GATCCACTAACAACCCA	8	-	319

MPSS signatures identified within the sense-strand sequence of CTG1027770. Uppercase text indicates the predicted ORF; lowercase indicates the predicted UTRs. Sense signatures are highlighted in blue. The position of the most abundant signature (#2) is consistent with the most-3' *Dpn*II site, which is the position predicted to be measured by MPSS. Other signatures may result from signatures resulting from other transcripts, alternative polyadenylation or incomplete digestion during the construction of the cDNA library.

```
gggggaagctctctcaatcactgctatccacccaaaccctagctctaaccctacccaagATGAATAGGGAGAAGCTTA
TGAAGATGGCTGGTGCAGTTCGCACCGGTGGAAAGGGTAGCATGAGAAGAAAGAAGAAGGCTGTTCACAAAACAACCA
CAACTGATGACAAAAGGCTTCAAAGCACCCTAAAGAGAATAGGAGTCAATGCCATACCTGCTATTGAGGAGGTCAACA
TCTTTAAAGATGATGTGGTTATTCAGTTTTTAAACCCCAAAGTTCAAGCCTCTATAGCCGCAAACACTTGGGTTGTTA
GTGGATCTCCTCAAACAAAGAAATTGCAGGACATTCTTCCTGGAATTATCAACCAGTTGGGACCAGATAACTTGGACA
ACCTGAGGAAGCTGGCTGAGCAGTTCCAGAAGCAGGCACCTGGTGCTGGTGCAGCAGCAGCCCAAGACGATGATGATG
ATGAGGTCCCCGATCTTGTTGCTGGGCAGACCTTTGAAGCTGCAGCAGAAGAGGGTCACACTGCTtgagagagttttt
agagggttttgtgccattacttatatttccaattgagagttttatcctttatttcgtttaaccaagtgtttcatttta
tgctctccttgtgacaccatactagacagttgtatgctcctgcctgaagactttactattttccaaatgcctgatttg
gtgttgtctagttaccttaaaagcattttctcagaccatccaattcctttttactttcatggttttgcctttatcagag
gaattcattttctccct
```
