# Supplementary material for: Comparative expression profiling in grape (Vitis vinifera) berries derived from frequency analysis of ESTs and MPSS signatures
Source: BMC Plant Biol. 2008 May 12;8:53. doi: 10.1186/1471-2229-8-53 (PMC2423195; doi:10.1186/1471-2229-8-53)
Supplement: Additional file 4 — Example of a grape EST contigs matched by multiple MPSS signatures. One of the EST contigs with 3 MPSS signatures matches is shown. This contig (CTG1027770) has similarity to "putative transcription factor BTF3-like mRNA". Panel A shows all the MPSS signatures identified in this contig, with the abundance level and its coordinate on the contig. Panel B displays the sequence of this contig and all the sense MPSS signatures from panel A are indicated in blue. Uppercase letters indicate the predicted ORF, while lowercase letters indicate the predicted UTRs. The position of the most abundant signature (#2) is consistent with the most-3' DpnII site, the position measured by MPSS. Other signatures may result from signatures resulting from other transcripts, alternative polyadenylation or incomplete digestion during the construction of the MPSS library. [file 1471-2229-8-53-S4.pdf]

Additional File 4: Occurrence of identical tags in related and unrelated contigs.

>CTG1027103 (F20O9.210 12S cruciferin seed storage protein, At4g28520, 1.5732e-45)
GTTTCTGTCCTCGACGTCAGCAATGAGGCCAACCAACTTGATTTCCAACCCAGAAGATTCTATCTTGCCGGCGACCCA
CAAAATGAATTTCAGCAGCGACAGCAACAACCACAAGAACAACAACAAGGAAGCGAGGGCCAGCAGCAACANCNNGAA
GGGGGCGGCAGTGAGGGCAGATNNNNNNNNNGNNNNGNAGACAACATCTTCAGTGGTTTCGATGCACAGCAGTTGGCT
GAGGCATTTAACGTGGATGTCCAGCTCATAAGGAAGCTTCAGGGCCAAAATGACAGGAGGGGCAACATAGTGAGAGTG
GAGGGAGGCCTACAGGCACTGTTACCACCAAGGGGACAACAGGAGCGAGGAGAGCAACAGCAAGATCACCTCCATGCA
CGCGGAAATGGCTATGAAGAGACCATATGCAGCTTGAGGCTCAAACAAAACATCGGTGATCCATGGAGAGCAGATGTC
TACACACCTAGAGGTGGACACAGGAGCAGCGTCACCGGCTATGATCTTCCAGTTCTCCAAAAGCTTGTTAAATTAAGT
GCTCATAAAGGACGTCTATATCAGGGCGCATTGGGGCTACCATACTACAACGTGAATGCCAATAGTGTAATCTACGCT
ATAAGAGGCAGTGCCAGGATTCAAGTAGTGCAACAGCAGNNNCAGACAGTGGCCAATGAGGAAGTCCAGCAGGGTCAG
GTGTTGGTTATTCCACAGAACTTCGCTGCGTTGATAAAAGCAAGGGATTCAGGCTTTGAGTATGTGGC**GATCAAGACT**
GATGAAAATGCCATGATTAACACTTTAGCNNNCAATCTTTCGCTGAT

>CTG1027117 (F20O9.210 12S cruciferin seed storage protein, At4g28520, 1.15902e-40)
ACATATGCCCGGGATTTCGGCCATNACNNNNNGGGGGGGACAACAGGAGCGAGGAGAGCAACAGCAAGATCACCTCCA
TGCACGCGGAAATGGCTATGAAGAGACCATATGCAGCTTGAGGCTCAAACAAAACATCGGTGATCCATGGAGAGCAGA
TGTCTACACACCTAGAGGTGGACACAGGAGCAGCGTCACCGGCTATGATCTTCCAGTTCTCCAAAAGCTTGTTAAATT
AAGTGCTCATAAAGGACGTCTATATCAGGGCGCATTGGTGCTACCATACTACAACGTGAATGCCAATAGTGTAATCTA
CGCTATAAGAGGCAGTGGTACAACGCAGAGTGGCCATTACGGCCGGGGTGCAACAGCAGGGCCAGACAGTGGCCAATG
AGGAAGTCCAGCAGGGTCAGGTGTTGGTTATTCCACAGAACTTCGCTGCGTTGATAAAAGCAAGGGATTCAGGCTTTG
AGTATGTGGC**GATCAAGACTGATGAAA**ATGCCATGATTAACACTTTAGCCGGCAATCTTTCGCTGATGCGGGCTATGC
CAGTCCAAGTCATAGCCAGCGCCTACCAGGCGTCCAATAACGAGGCTAAGCAGCTGAAGCATAACAGGCAGGAGAGCA
CTATAGGGGCTCCGGGAAGTTCTAGGTCTGAGTAGAGGGCCTCTGCTTAATGCGTCAACTTTGTTTTATCAGGTTGTT
GTATAGGTTTGGAAAATAATAAGANNNCCATATTACTTGGCTTTTTTGTC

>CTG1027135 (MLN1.4 legumin-like protein, At5g44120, 3.62753e-86)
CAGCTTAGTAGTCTTATCTGCAGTGTTACCATGGCTAAGAGTCTTTGTTGCCTTTTGCTTTGCTTTCTTATTTTCTTC
AATTACTGCTCGGCTCGGCAGCAAAAGTCTAGGCAGCAAAATGAGTGCAGCATCTCTAGACTCACTGCTCAGAAACCT
TCCAACCGGATCCAGTCAGAGGCCGGTGTGACCGAGGTCTTTGATCATAACAATGAGCAATTCCAGTGCGCGGGTGTG
GCTGTTGTTCGCTATACTATTGAACCCAACGGCTTGCTTTTGCCTTCCTACGTCAATGCGCCTCAACTCTTGTACTTC
GTCCAAGGTAGAGGCTTGCAGGGAATCCTGATCAGTGGTTGTCCTGAAACCTTCCAATCATTCCAGGAATCTCAGGAA
GGGCATCAAGGAAGAGAACAGGAAGGGCAGCAAGGACAACAGGGACAACAAGGCCAGCAAGGACAACAAGGCCAGCAA
GGACAACAAGGCCAGCAATTCCTTGGAGACCAACATCAGAAGATCCGAGAGGTCGAAGAGGGTGATGCTTTTGCAGTG
CCCACCGGATTTGGGCATTATATCTATAACAATGGTAACAGACAGCTTGTTGTAGTTTCTGTCCTCGACGTCAGCAAT
GAGGCCAACCAACTTGATTTCCAACCCAGAAGATTCTATCTTGCCGGCAACCCACAAAATGAATTCCAGCAGCAACAG
CAACAACAACAAGGAAGCGAGGGCCAGCAGCAACAGCAAGAAGGGGGCGGCAGTGAGGGTAGAGGGCAAGAGAGTTCC
GGCGACAACATCTTCAGTGGTTTCGATGCACAGCAGTTGGCTGAGGCATTTAACGTGGATGTCCAGCTCATAAGGAAG
CTTCAGGGCCAAAATGACAGGAGGGGCAACATAGTGAGAGTGGAGGGAGGCCTACAGGCACTGTTACCACCGAGGGGA
CAACAGGAGCGAGGAGAGCAACAGCAAGATCACCTCCATGCACGCGGAAATGGCTATGAAGAGACCATATGCAGCTTG
AGGCTCAAACAAAACATCGGTGATCCATGGAGAGCAGATGTCTACACACCTAGAGGTGGACACAGGAGCAGCGTCACC
GGCTATGATCTTCCAGTTCTCCAAAAGCTTGTTAAATTAAGTGCTCATAAAGGACGTCTATATCAGGGCGCATTGGTG
CTACCATACTACAACGTGAATGCCAATAGTGTAATCTACGCTATAAGAGGCAGTGCCAGGATTCAAGTAGTGCAACAG
CAGGGCCAGACAGTGGCCAATGAGGAAGTCCAGCAGGGTCAGGTGTTGGTTATTCCACAGAACTTCGCTGCGTTGATA
AAAGCAAGGGATTCAGGCTTTGAGTATGTGGC**GATCAAGACTGATGAAA**ATGCCATGATTAACACTTTAGCCGGCAAT
CTTTCGCTGATGCGGGCTATGCCAGTCCAAGTCATAGCCAGCGCCTACCAGGCGTCCAATAACGAGGCTAAGCAGCTG
AAGCATAACAGGCAGGAGAGCACTATAGGGGCTCCGGGAAGTTCTAGGTCTGAGTAGAGGGCCTCTGCTTAATGCATC
AACTTTGTTTTATCAGGTTGTTGTATAGGTTTGGAAAATAATAAGAGGCCATATTACTTGGCTTTTTTGTACCCCATG
ATGTACAATGAGAGTTGAGTTTTTGCTCTTTAATAATAATAATAATAACATCAATAATGCTTATGCAGTAAA
