# Supplementary material for: Comparative expression profiling in grape (Vitis vinifera) berries derived from frequency analysis of ESTs and MPSS signatures
Source: BMC Plant Biol. 2008 May 12;8:53. doi: 10.1186/1471-2229-8-53 (PMC2423195; doi:10.1186/1471-2229-8-53)
Supplement: Additional file 5 — Occurrence of identical MPSS signatures in related and unrelated contigs. The example of MPSS signatures with multiple hits to EST contigs is shown. In this particular example, the MPSS signature "GATCAAGACTGATGAAA" (displayed in red) was identified in three EST contigs where two of them have the same annotation and the third is different. The most closely related Arabidopsis homolog along with its BLAST expected value is list at the beginning of each coding sequence. [file 1471-2229-8-53-S5.pdf]

Additional File 4: Occurrence of identical tags in related and unrelated contigs.

>CTG1027103 (F20O9.210 12S cruciferin seed storage protein, At4g28520, 1.5732e-45)
GTTTCTGTCCTCGACGTCAGCAATGAGGCCAACCAACTTGATTTCCAACCCAGAAGATTCTATCTTGCCGGCGACCCA
CAAAATGAATTTCAGCAGCGACAGCAACAACCACAAGAACAACAACAAGGAAGCGAGGGCCAGCAGCAACANCNNGAA
GGGGGCGGCAGTGAGGGCAGATNNNNNNNNNGNNNNGNAGACAACATCTTCAGTGGTTTCGATGCACAGCAGTTGGCT
GAGGCATTTAACGTGGATGTCCAGCTCATAAGGAAGCTTCAGGGCCAAAATGACAGGAGGGGCAACATAGTGAGAGTG
GAGGGAGGCCTACAGGCACTGTTACCACCAAGGGGACAACAGGAGCGAGGAGAGCAACAGCAAGATCACCTCCATGCA
CGCGGAAATGGCTATGAAGAGACCATATGCAGCTTGAGGCTCAAACAAAACATCGGTGATCCATGGAGAGCAGATGTC
TACACACCTAGAGGTGGACACAGGAGCAGCGTCACCGGCTATGATCTTCCAGTTCTCCAAAAGCTTGTTAAATTAAGT
GCTCATAAAGGACGTCTATATCAGGGCGCATTGGGGCTACCATACTACAACGTGAATGCCAATAGTGTAATCTACGCT
ATAAGAGGCAGTGCCAGGATTCAAGTAGTGCAACAGCAGNNNCAGACAGTGGCCAATGAGGAAGTCCAGCAGGGTCAG
GTGTTGGTTATTCCACAGAACTTCGCTGCGTTGATAAAAGCAAGGGATTCAGGCTTTGAGTATGTGGC**GATCAAGACT**
GATGAAAATGCCATGATTAACACTTTAGCNNNCAATCTTTCGCTGAT

>CTG1027117 (F20O9.210 12S cruciferin seed storage protein, At4g28520, 1.15902e-40)
ACATATGCCCGGGATTTCGGCCATNACNNNNNGGGGGGGACAACAGGAGCGAGGAGAGCAACAGCAAGATCACCTCCA
TGCACGCGGAAATGGCTATGAAGAGACCATATGCAGCTTGAGGCTCAAACAAAACATCGGTGATCCATGGAGAGCAGA
TGTCTACACACCTAGAGGTGGACACAGGAGCAGCGTCACCGGCTATGATCTTCCAGTTCTCCAAAAGCTTGTTAAATT
AAGTGCTCATAAAGGACGTCTATATCAGGGCGCATTGGTGCTACCATACTACAACGTGAATGCCAATAGTGTAATCTA
CGCTATAAGAGGCAGTGGTACAACGCAGAGTGGCCATTACGGCCGGGGTGCAACAGCAGGGCCAGACAGTGGCCAATG
AGGAAGTCCAGCAGGGTCAGGTGTTGGTTATTCCACAGAACTTCGCTGCGTTGATAAAAGCAAGGGATTCAGGCTTTG
AGTATGTGGC**GATCAAGACTGATGAAA**ATGCCATGATTAACACTTTAGCCGGCAATCTTTCGCTGATGCGGGCTATGC
CAGTCCAAGTCATAGCCAGCGCCTACCAGGCGTCCAATAACGAGGCTAAGCAGCTGAAGCATAACAGGCAGGAGAGCA
CTATAGGGGCTCCGGGAAGTTCTAGGTCTGAGTAGAGGGCCTCTGCTTAATGCGTCAACTTTGTTTTATCAGGTTGTT
GTATAGGTTTGGAAAATAATAAGANNNCCATATTACTTGGCTTTTTTGTC

>CTG1027135 (MLN1.4 legumin-like protein, At5g44120, 3.62753e-86)
CAGCTTAGTAGTCTTATCTGCAGTGTTACCATGGCTAAGAGTCTTTGTTGCCTTTTGCTTTGCTTTCTTATTTTCTTC
AATTACTGCTCGGCTCGGCAGCAAAAGTCTAGGCAGCAAAATGAGTGCAGCATCTCTAGACTCACTGCTCAGAAACCT
TCCAACCGGATCCAGTCAGAGGCCGGTGTGACCGAGGTCTTTGATCATAACAATGAGCAATTCCAGTGCGCGGGTGTG
GCTGTTGTTCGCTATACTATTGAACCCAACGGCTTGCTTTTGCCTTCCTACGTCAATGCGCCTCAACTCTTGTACTTC
GTCCAAGGTAGAGGCTTGCAGGGAATCCTGATCAGTGGTTGTCCTGAAACCTTCCAATCATTCCAGGAATCTCAGGAA
GGGCATCAAGGAAGAGAACAGGAAGGGCAGCAAGGACAACAGGGACAACAAGGCCAGCAAGGACAACAAGGCCAGCAA
GGACAACAAGGCCAGCAATTCCTTGGAGACCAACATCAGAAGATCCGAGAGGTCGAAGAGGGTGATGCTTTTGCAGTG
CCCACCGGATTTGGGCATTATATCTATAACAATGGTAACAGACAGCTTGTTGTAGTTTCTGTCCTCGACGTCAGCAAT
GAGGCCAACCAACTTGATTTCCAACCCAGAAGATTCTATCTTGCCGGCAACCCACAAAATGAATTCCAGCAGCAACAG
CAACAACAACAAGGAAGCGAGGGCCAGCAGCAACAGCAAGAAGGGGGCGGCAGTGAGGGTAGAGGGCAAGAGAGTTCC
GGCGACAACATCTTCAGTGGTTTCGATGCACAGCAGTTGGCTGAGGCATTTAACGTGGATGTCCAGCTCATAAGGAAG
CTTCAGGGCCAAAATGACAGGAGGGGCAACATAGTGAGAGTGGAGGGAGGCCTACAGGCACTGTTACCACCGAGGGGA
CAACAGGAGCGAGGAGAGCAACAGCAAGATCACCTCCATGCACGCGGAAATGGCTATGAAGAGACCATATGCAGCTTG
AGGCTCAAACAAAACATCGGTGATCCATGGAGAGCAGATGTCTACACACCTAGAGGTGGACACAGGAGCAGCGTCACC
GGCTATGATCTTCCAGTTCTCCAAAAGCTTGTTAAATTAAGTGCTCATAAAGGACGTCTATATCAGGGCGCATTGGTG
CTACCATACTACAACGTGAATGCCAATAGTGTAATCTACGCTATAAGAGGCAGTGCCAGGATTCAAGTAGTGCAACAG
CAGGGCCAGACAGTGGCCAATGAGGAAGTCCAGCAGGGTCAGGTGTTGGTTATTCCACAGAACTTCGCTGCGTTGATA
AAAGCAAGGGATTCAGGCTTTGAGTATGTGGC**GATCAAGACTGATGAAA**ATGCCATGATTAACACTTTAGCCGGCAAT
CTTTCGCTGATGCGGGCTATGCCAGTCCAAGTCATAGCCAGCGCCTACCAGGCGTCCAATAACGAGGCTAAGCAGCTG
AAGCATAACAGGCAGGAGAGCACTATAGGGGCTCCGGGAAGTTCTAGGTCTGAGTAGAGGGCCTCTGCTTAATGCATC
AACTTTGTTTTATCAGGTTGTTGTATAGGTTTGGAAAATAATAAGAGGCCATATTACTTGGCTTTTTTGTACCCCATG
ATGTACAATGAGAGTTGAGTTTTTGCTCTTTAATAATAATAATAATAACATCAATAATGCTTATGCAGTAAA
